# Transcription Control Mechanisms for Plant Stress Responses

**DOI:** 10.3390/ijms24076824

**Published:** 2023-04-06

**Authors:** Jong-Joo Cheong

**Affiliations:** Center for Food and Bioconvergence, Seoul National University, Seoul 08826, Republic of Korea; cheongjj@snu.ac.kr

Plants have their roots fixed in the soil, so they are unable to escape from adverse environments. Thus, they must deal with various biotic and abiotic stresses by regulating gene expression to induce cellular tolerance responses. To initiate gene transcription correctly, RNA polymerase II must come together with several general transcription factors on a core promoter to form a large multiprotein–DNA preinitiation complex. In contrast, gene-specific transcription factors recognize specific upstream *cis*-acting DNA elements of their target genes to activate transcription. In many cases, additional coactivators or regulators, including mediator complexes, connect the gene-specific transcription factors and transcription machinery (e.g., general transcription factors), thereby transducing signals from the *cis*-acting regulatory sequences to the core promoter to modulate target gene expression.

In eukaryotic cells, genomic DNA is packed into the nucleus by wrapping it around histones to form nucleosomes, the basic unit of chromatin structure. To initiate the transcription of a gene, the DNA–histone interaction in the surrounding chromatin must be relaxed to enable access by transcriptional activators and RNA polymerase. Gene expression is accompanied by chromatin remodeling (e.g., histone modification and promoter DNA methylation). In addition, small and long non-coding RNAs modulate gene expression at the transcriptional and posttranscriptional levels. The epigenetic changes can be transmitted to newly developed cells during vegetative growth and even inherited by the progeny. Thus, plants pre-exposed to stressful conditions often grow better under subsequent stressful conditions in the same generation and in the next generation of the plants.

This Special Issue entitled ‘Transcription control mechanisms for plant stress responses’ in the *International Journal of Molecular Sciences* includes a total of five contributions. Three original articles describe the discovery and characterization of gene-specific transcription factors from cotton, strawberry, and iris [1,2,3]. In addition, the review article by Chen et al. [4] provides comprehensive information about mediator complexes, which connect gene-specific transcription factors and general transcription factors to control target gene expression. Furthermore, Nguyen et al. [5] reviewed recent findings on the epigenetic mechanism underlying transcriptional stress memory and the transgenerational inheritance of drought tolerance in plants.

Liu et al. [1] identified a nucleus-localized cotton-gene-specific transcription factor, GhHSFB2a, which is crucial for resistance to the soil-borne fungus *Verticillium dahliae*. In that study, a *cis*-acting element-FC-rich motif was found to be responsible for the defense response to *V. dahliae*. Upon inoculation with the fungus, the *GhHSFB2a* gene transcripts accumulated in the roots, stems, and leaves. Ectopic expression of *GhHSFB2a* in Arabidopsis enhanced resistance to *V. dahlia*, while virus-induced gene silencing of the gene significantly impaired cotton resistance. Transcriptome analyses revealed that GhHSFB2a participates in cotton disease resistance by regulating abscisic acid (ABA) and ethylene signaling, linoleic acid metabolism, and the phenylpropanoid pathway.

Li et al. [2] identified 126 C2H2-type zinc finger proteins (C2H2-ZFPs) from a database of the strawberry genome. Among them, 46 members were identified as subclass C1-2i and further classified into five groups according to their phylogenetic relationships. Members in each group have similar physicochemical properties and motif compositions. Analyses of their conserved domains and gene structures indicated that the C1-2i members were subjected to purifying selection during evolution. In particular, the gene encoding FaZAT10, a typical C2H2-ZFP belonging to subclass C1-2i, was expressed mainly in the roots and induced by ABA treatment and under drought, saline, or low-temperature conditions, implying its potential role in abiotic stress tolerance responses.

Xu et al. [3] investigated the dormancy induction process in two closely related iris species exhibiting an eco-dormancy-only process, the evergreen *Iris japonica* Thunb and the deciduous *Iris tectorum* Maxim, by evaluating their morphological and physiological characteristics to ensure their developmental status. Under artificial dormancy-inducing conditions, the deciduous iris entered into dormancy earlier than the evergreen iris. The expression patterns of the dormancy marker genes involved in key pathways of winter dormancy suggested important roles for phytohormones and carbohydrates in coordinating growth and stress responses during dormancy induction in both iris species. In particular, it appears that dormancy-related MADS-box genes and Snf1-related protein kinases (SnRKs) represent a bridge between carbohydrate and phytohormone interactions during dormancy. These findings allude to a mechanistic model for investigating the key factors contributing to later dormancy in the evergreen iris relative to the deciduous iris. This study provides a basis for future research into the molecular mechanisms in perennials that exclusively exhibit eco-dormancy in the transition zone between temperate and subtropical climates, together with a reference for postharvest storage, shipping, and breeding.

Mediator complexes transmit signals delivered by gene-specific transcription factors to the transcription machinery assembled at promoters to control transcription initiation. Chen et al. [4] present a comprehensive review of recent advances in the study of the structure and function of plant mediator complexes. The authors reviewed studies on the roles of Mediator complexes in plant adaptive responses to a wide spectrum of environmental stresses. The focus was on ABA-mediated stress responses, other responses to abiotic stresses, the signaling of jasmonic acid as a stress-responsive hormone, broad plant defenses against biotic attacks, and the regulation of phenylpropanoid biosynthesis. Despite substantial progress in this field of study, the authors point out that there are still many important questions about the roles of mediator complexes in the regulation of target gene transcription, functional specificity, and the molecular mechanisms by which they are recruited to target gene promoters. Most published research on plant mediator complexes has been carried out in Arabidopsis; therefore, the authors emphasize the need to expand this research to other crop plants to improve agronomical strategies.

Nguyen et al. [5] reviewed recent research on the epigenetic molecular mechanisms underlying transcriptional drought-stress memory in plants, summarizing chromatin architecture changes (DNA methylation, histone modification, and chromatin loops) that maintain the altered gene expression patterns caused by an initial stressor during the stress memory response. Reflecting on observations from studies of invertebrates (*Drosophila* and *Caenorhabditis elegans*) and the process of flowering in plants, the authors propose a hypothetical model for the mechanism of mitotic transmission in transcriptional memory. In this model, epigenetic marks, including modified histones (e.g., H3K27me3 and H3K4me3) and chromatin regulators (e.g., polycomb group (PcG) protein complexes and trithorax group (TrxG) complexes) remain bound to chromatin loops formed in response to stress and are transmitted through mitosis. Certain transcription factors and chromatin remodeling complexes remain in close proximity to the chromatin of a memory locus, establishing a subsequent transcriptional status (e.g., silencing or activation). However, there is little evidence to support the notion that chromatin architecture formation contributes to the memory of drought-stress tolerance in plants. Therefore, this speculative model should be verified in future studies of the mechanisms of drought-stress memory.

For transgenerational inheritance, possible roles for DNA methylation are discussed [5], with reports describing either positive or negative observations. In contrast to mammals, DNA methylation in flowering plants may not be completely erased from the germlines and is thus maintained during reproduction. Based on invertebrate developmental processes and the plant flowering process, it is suggested that modified histones and other core chromatin components survive the passage of replication forks during meiosis. Verification of the relevance of these mechanisms to the transgenerational inheritance of drought-stress tolerance requires further investigation.

The identification of specific transcription factors in a wide range of agronomically important crops, and elucidation of the molecular mechanisms underlying environmental stress tolerance, will facilitate the breeding of crops that are able to withstand global climate change more effectively. In particular, the principles and mechanisms underlying cellular memory should be further exploited to improve agronomic technology and crop breeding because epimutations can be artificially induced more rapidly than through traditional genetic manipulation.
